# Heart failure–related genes associated with oxidative stress and the immune landscape in lung cancer

**DOI:** 10.3389/fimmu.2023.1167446

**Published:** 2023-05-18

**Authors:** Ruoshu Duan, Kangli Ye, Yangni Li, Yujing Sun, Jiahong Zhu, Jingjing Ren

**Affiliations:** Department of General Practice, The First Affiliated Hospital, Zhejiang University School of Medicine, Hangzhou, Zhejiang, China

**Keywords:** heart failure, lung cancer, comorbidity, immune infiltration, oxidative stress

## Abstract

**Background:**

Lung cancer is a common comorbidity of heart failure (HF). The early identification of the risk factors for lung cancer in patients with HF is crucial to early diagnosis and prognosis. Furthermore, oxidative stress and immune responses are the two critical biological processes shared by HF and lung cancer. Therefore, our study aimed to select the core genes in HF and then investigate the potential mechanisms underlying HF and lung cancer, including oxidative stress and immune responses through the selected genes.

**Methods:**

Differentially expressed genes (DEGs) were analyzed for HF using datasets extracted from the Gene Expression Omnibus database. Functional enrichment analysis was subsequently performed. Next, weighted gene co-expression network analysis was performed to select the core gene modules. Support vector machine models, the random forest method, and the least absolute shrinkage and selection operator (LASSO) algorithm were applied to construct a multigene signature. The diagnostic values of the signature genes were measured using receiver operating characteristic curves. Functional analysis of the signature genes and immune landscape was performed using single-sample gene set enrichment analysis. Finally, the oxidative stress–related genes in these signature genes were identified and validated *in vitro* in lung cancer cell lines.

**Results:**

The DEGs in the GSE57338 dataset were screened, and this dataset was then clustered into six modules using weighted gene co-expression network analysis; MEblue was significantly associated with HF (cor = *−*0.72, p < 0.001). Signature genes including extracellular matrix protein 2 (ECM2), methyltransferase-like 7B (METTL7B), meiosis-specific nuclear structural 1 (MNS1), and secreted frizzled-related protein 4 (SFRP4) were selected using support vector machine models, the LASSO algorithm, and the random forest method. The respective areas under the curve of the receiver operating characteristic curves of ECM2, METTL7B, MNS1, and SFRP4 were 0.939, 0.854, 0.941, and 0.926, respectively. Single-sample gene set enrichment analysis revealed significant differences in the immune landscape of the patients with HF and healthy subjects. Functional analysis also suggested that these signature genes may be involved in oxidative stress. In particular, METTL7B was highly expressed in lung cancer cell lines. Meanwhile, the correlation between METTL7B and oxidative stress was further verified using flow cytometry.

**Conclusion:**

We identified that ECM2, METTL7B, MNS1, and SFRP4 exhibit remarkable diagnostic performance in patients with HF. Of note, METTL7B may be involved in the co-occurrence of HF and lung cancer by affecting the oxidative stress immune responses.

## 1 Introduction

Heart failure (HF) is a devastating clinical syndrome characterized by symptoms and/or signs caused by structural and/or functional impairments in ventricular filling or blood ejection (1). The global prevalence of HF across patients of all ages is 1.3% but can be as high as 8.3% in people aged >50 years; moreover, the incidence of HF increases with age (2). HF can be classified on the basis of the onset type (acute vs. chronic), the affected ventricle (left vs. right), and the type of circulation affected (systolic vs. diastolic) (3). Hypertension, obesity, prediabetes, diabetes, and atherosclerotic cardiovascular disease are the main risk factors for HF (4). The leading causes of HF include ischemic heart disease and myocardial infarction, hypertension, and valvular heart disease (1). The pathophysiological mechanisms of HF involve comprehensive biological processes, including ischemia (5), mechanical stress (6, 7), autoimmune disease (8), microbial infections (9), and genetic diseases.

In western countries, patients with HF have an average of five different diseases simultaneously, with cancer being one of the main causes of non-cardiovascular death in patients with HF (10). In addition, patients with HF have a significantly increased risk of cancer (approximately 76%) as compared with those without HF (10–12). Banke et al. reported that lung cancer was one of the most common malignant diagnoses in the HF cohort, with a proportion of approximately 15.7% (13). The prevalence of HF and cancer has increased with the aging population and advancing medical technology. The co-existence of cancer in patients with HF is becoming more common owing to the similar pathogenesis of and risk factors for the two diseases, including inflammation, angiogenesis, clonal hematopoiesis, metabolic remodeling and extracellular matrix (ECM), and stromal cell infiltration (14, 15). In particular, the risk of respiratory cancer is increased by 91% among patients with HF (10). The causes of the co-existence of HF and lung cancer are not entirely clear; however, research has suggested that the two diseases share some risk factors, such as smoking, advanced age, high blood pressure, diabetes, and obesity (16–19). Moreover, some lung cancer treatments, such as radiation and chemotherapy, can also damage the heart, thereby increasing the risk of HF (20).

To investigate the correlation between HF and lung cancer, first, we aimed to use multiple bioinformatic approaches to identify the signature genes in HF. Next, we performed enrichment pathway analyses to investigate the correlations in these genes and the roles that they play in lung cancer. Our results will provide clinicians with new insights into the diagnosis and treatment of lung cancer.

## 2 Method and materials

### 2.1 Data acquisition and the identification of differentially expressed gene

The GSE57338 dataset related to HF was downloaded from the Gene Expression Omnibus database (http://www.ncbi.nlm.nih.gov/geo/). The differentially expressed genes (DEGs) between patients with HF and healthy subjects were selected using the R software’s limma package (21) (criteria: p < 0.05 and |log fold change (FC)| > 1), and the results were visualized as volcano plots and heatmaps.

### 2.2 Gene Ontology and Kyoto Encyclopedia of Genes and Genomes analysis

The enrichment of the functions of the selected DEGs was performed using Gene Ontology (GO) and Kyoto Encyclopedia of Genes and Genomes (KEGG) analysis with the cluster profiler package in R (22).

### 2.3 Weighted gene co-expression network analysis

A weighted gene co-expression network analysis (WGCNA) (23) was performed using the gene expression profiles from the GSE57338 dataset to explore the hub genes based on the soft threshold power chosen by the pickSoftThreshold function. The dynamic tree-cutting method was then used to identify the co-expressed gene modules. The Pearson correlation coefficients between the module eigengenes and HF were evaluated to acquire the hub module.

### 2.4 Signature gene identification

First, the candidate hub genes were identified through the intersection of the DEGs and key module genes. Second, the signature genes were selected according to calculations conducted using support vector machine (SVM) models, the least absolute shrinkage and selection operator (LASSO) algorithm (24), and the random forest (RF) model (25). Finally, the area under the receiver operating characteristic (AUC-ROC) curve was used to evaluate the diagnostic efficiency of each selected signature gene. An AUC > 0.7 indicated a favorable diagnostic performance.

### 2.5 The immune landscape and functional analysis

The single-sample gene set enrichment analysis (ssGSEA) algorithm was used with R packages (GSVA and GSEABase) (26) to comprehensively assess the immunologic characteristics between the patients with HF and healthy subjects. The marker genes of different immune cells were derived from previous studies (27) and are listed in **Table S1**. Next, functional analysis of the signature genes was performed using the ssGSEA algorithm.

### 2.6 Identification of oxidative stress–related genes

The oxidative stress–related genes were selected on the basis of the background information obtained from GeneGards (https://www.genecards.org/). A total of 10,022 oxidative stress–related genes were screened.

### 2.7 Cell culture

The human lung cancer cell lines A549 and PC9 and the normal human lung epithelial cell line BEAS2B were obtained from the American Type Culture Collection (Manassas, VA, USA). A549 was cultured in Roswell Park Memorial Institute-1640 medium, whereas PC9 and BEAS2B were cultured in Dulbecco’s Modified Eagle Medium supplemented with 10% fetal bovine serum at 37°C in an incubator with 5% CO_2_.

### 2.8 Quantitative polymerase chain reaction and Western blotting

Total RNA was isolated from the cells using the RNA-Quick Purification Kit (ESscience, RN001, China). A reverse transcription kit (Vazyme, R333-01, China) was used to reverse-transcribe RNA into cDNA, and real-time fluorescence quantitative PCR (Vazyme, Q711-02, China) was performed using Glyceraldehyde-3-phosphate dehydrogenase (GAPDH) as the control. Finally, the relative mRNA expression of METTL7B was calculated using the 2^−ΔΔCt^ technique to assess METTL7B expression in lung cancer cell lines and the normal human lung epithelial cell line. The sequences of the PCR primers used in this study are listed in the **Supplementary Information** (**Table S2**).

The cell samples were collected, and total protein was extracted according to the manufacturer’s instructions. The BCA Protein Assay Kit (Beyotime Biotechnology, China) was used to measure the protein concentration. The proteins were separated by electrophoresis and then transferred from the gel onto polyvinylidene difluoride membranes (Merck Millipore, USA, cat. no. IPVH00010). Blocking was performed using 5% non-fat dry milk powder (room temperature, 1 h). Incubation with the following primary antibodies was performed overnight at 4°C: anti-METTL7B (1:1,000 dilution; Proteintech, cat. no. 17001-1-AP) and anti–β-actin (1:1,000 dilution; Huabio, cat. no. ET1701-80). The membranes were then incubated with horseradish peroxidase–conjugated secondary antibodies (room temperature, 1 h), and the binding was detected using an enhanced chemiluminescence reagent (Sangon Biotech, China, cat. no. C510045-0100).

### 2.9 Plasmid siRNA transfection

For plasmid transfection, lipofectamine 3000 (Invitrogen, cat. no. L3000008) was used for the A549 and PC9 cells. For small interfering RNA (siRNA) transfection, lipofectamine RNAiMAX (Invitrogen, cat. no. 13778075) was used for the corresponding cells.

### 2.10 Oxidative stress measurement

The intracellular reactive oxygen species (ROS) levels in the cell samples were measured using the Reactive Oxygen Species Assay Kit (Beyotime Biotechnology, China), according to the manufacturer’s instructions. Cells with METTL7B overexpression or knockdown were incubated with 2′,7′-dichlorodihydrofluorescein diacetate for 30 min at 37°C and measured using CytoFLEX S (Beckman Coulter, CA, USA).

### 2.11 Statistical analysis

All statistical analyses were performed using R software (version 4.1.2). *p* < 0.05 was considered statistically significant. Unless specifically stated, all *p*-values were two-tailed. The research flow chart is shown in **Supplementary Figure S1**.

## 3 Results

### 3.1 DEGs and functional analysis of heart failure–related genes

**Figure 1:**
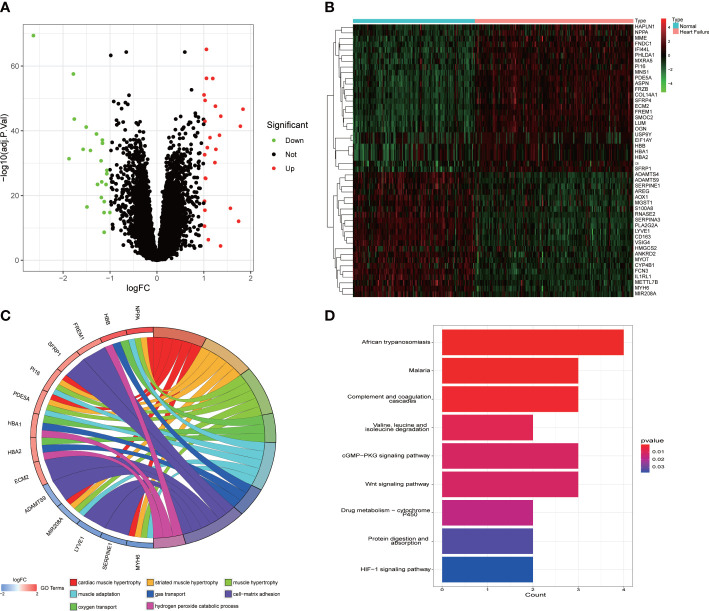
Identification of the DEGs in HF and functional enrichment analysis of the DEGs. **(A)** The green dots in the volcano map indicate the downregulated genes, the red dots indicate the upregulated genes, and the black dots indicate the genes with no significant differences. **(B)** The blue in the heat map represents the healthy samples, and the pink represents the HF samples. **(C)** The GO terms of the DEGs were enriched in the chord diagram. **(D)** KEGG analysis showing the functional enrichment pathways.

DEGs from the patients with HF and healthy controls were analyzed using the “limma” package. A total of 48 DEGs were finally screened, of which 26 were upregulated and 22 were downregulated (**Figure 1A**). The heatmap showed the DEGs between the patients with HF and healthy subjects (**Figure 1B**). The GO terms of the DEGs were enriched in the chord diagram (**Figure 1C**). As shown in the KEGG analysis, the top three enriched pathways were African trypanosomiasis, malaria, and complement and coagulation cascades (**Figure 1D**).

### 3.2 Construction of the WGCN

**Figure 2:**
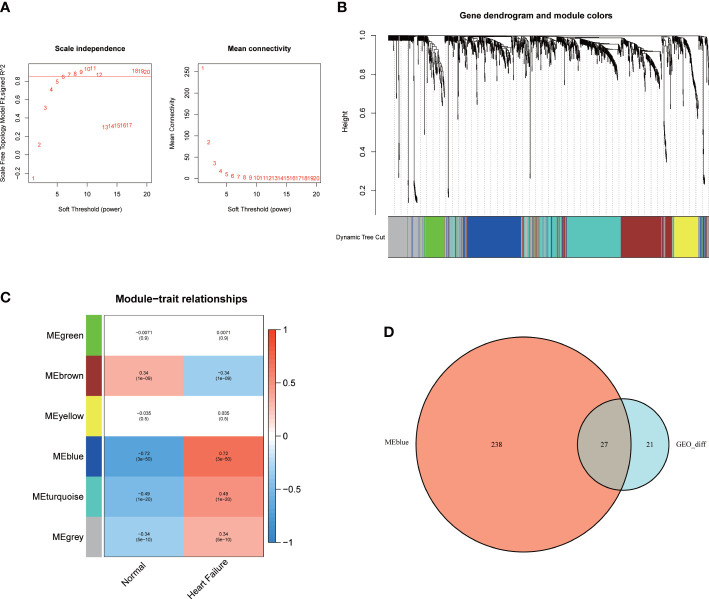
The WGCNA analysis and identification of candidate hub genes. **(A)** The soft threshold power and the mean connectivity of WGCNA. **(B)** The cluster dendrogram of WGCNA. **(C)** The clustered modules of WGCNA. **(D)** The venn diagram showed the interactions between the DEGs and genes in the MEblue module.

A co-expression network was established between the patients with HF and healthy subjects using the R package “WGCNA”. The soft threshold power was equivalent to 6 (**Figure 2A**). A cluster dendrogram was constructed (**Figure 2B**), and the data were clustered into six modules (**Figure 2C**). The relationship between the different modules and the patients with HF was assessed. The results suggested that MEblue, including 265 genes, was a pivotal module unrelated to the patients with HF (cor = 0.72, *p* < 0.001). The area with a total of 27 overlapping key DEGs was verified from the total DEGs and MEblue module–containing genes (**Figure 2D**).

### 3.3 Selection of the signature genes using machine algorithms

**Figure 3:**
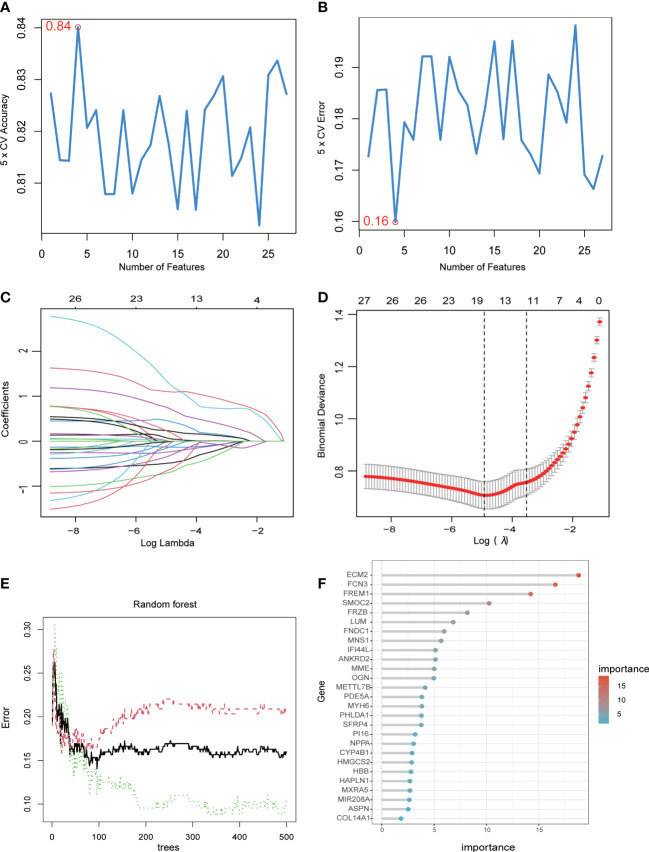
Machine learning algorithms determine signature genes. **(A)** The cross-validation accuracy of the SVM model. **(B)** The cross-validation error of the SVM model. **(C)** The LASSO algorithm showing variations in the size of coefficients for the parameters that shrank as the value of k penalty increased. **(D)** Penalty plot of the LASSO algorithm with error bars denoting standard errors. **(E)** The error rate confidence intervals of the RF model. **(F)** The relative importance of the genes in the RF model.

The abovementioned 27 key genes were further screened using machine algorithms. Consequentially, four genes were selected by the SVM model (**Figures 3A, B**), 17 by the LASSO algorithm (**Figures 3C, D**), and 19 by the RF method, with an importance of >3 (**Figures 3E, F**). Finally, their intersection was considered so as to obtain four genes: extracellular matrix protein 2 (ECM2), methyltransferase-like 7B (METTL7B), meiosis-specific nuclear structural 1 (MNS1), and secreted frizzled-related protein 4 (SFRP4) (**Figures 3E, F**).

### 3.4 Diagnostic efficacy of the signature genes in predicting HF

**Figure 4:**
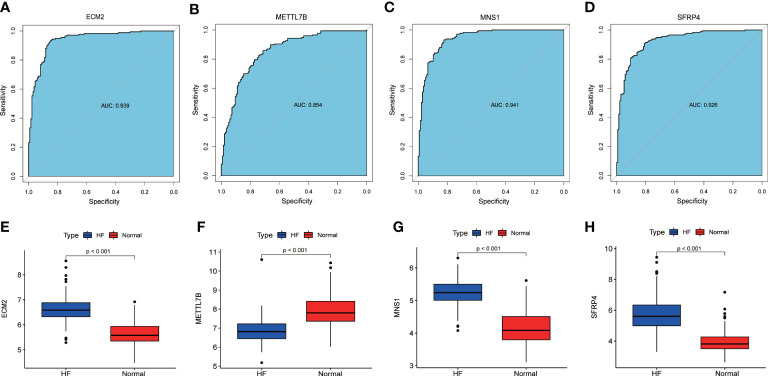
The ROC curve and expression of signature genes between the patients with HF and healthy subjects. **(A–D)** ROC curves of ECM2, METTL7B, MNS1, and SFRP4 between the patients with HF and healthy subjects. **(E–H)** Gene expression levels of ECM2, METTL7B, MNS1, and SFRP4 between the patients with HF and healthy subjects.

An AUC-ROC of 0.939 for ECM2, 0.854 for METTL7B, 0.941 for MNS1, and 0.926 for SFRP4 was obtained (**Figures 4A–D**). Furthermore, the expression levels of these signature genes were significantly higher in the patients with HF than in healthy subjects, indicating the potential roles of these signature genes in HF (**Figures 4E–H**).

### 3.5 Immune landscape

**Figure 5:**
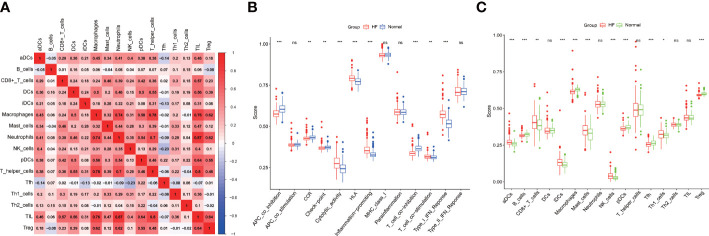
Immune landscape. **(A)** Immunological features. **(B)** Differences in the immune function between the patients with HF and healthy subjects. **(C)** Differences in the infiltration of immune cells between the patients with HF and healthy subjects. * p<0.05, ** p<0.01, *** p<0.001, “ns” means not significantly.

Regarding the relationships between the different immune cell subtypes, tumor-infiltrating lymphocytes and neutrophils had the strongest positive correlation (*r* = 0.87), and T follicular helper (Tfh) and natural killer cells had the strongest negative correlation (*r* = −0.23) (**Figure 5A**). The ssGSEA analysis showed that the patients with HF were significantly prone to cytolytic activity, human leukocyte antigen response, inflammation promotion, T-cell co-stimulation, and type I interferon response, whereas the healthy subjects showed antigen-presenting cell co-inhibition, cytokine receptor interaction, checkpoint, and T-cell co-inhibition (**Figure 5B**). The patients with HF presented low infiltration of B cells, macrophages, plasmacytoid dendritic cells, and Tfh and T regulatory (Treg) cells but high infiltration of aDCs, CD8^+^ T cells, immature dendritic cells mast cells, natural killer cells, and Th1 cells (**Figure 5C**).

### 3.6 ssGSEA analysis and the identification of oxidative stress–related genes

**Figure 6:**
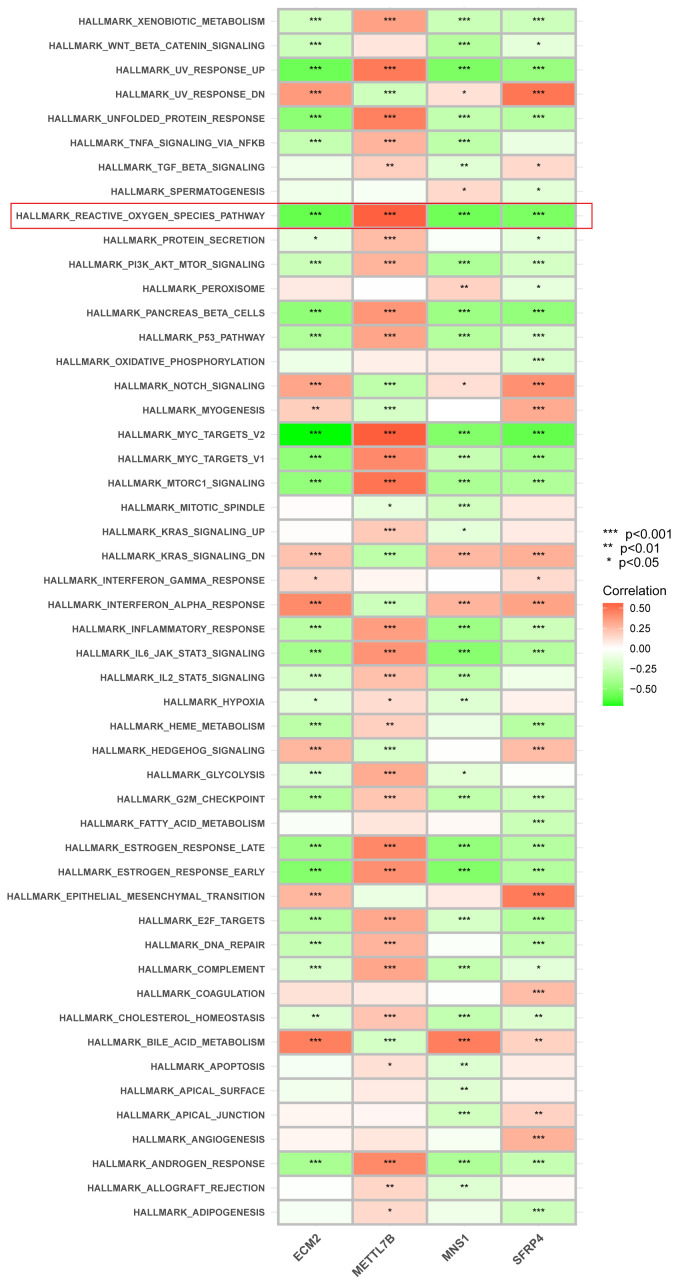
Correlation between each gene signature and pathway. ECM2, METTL7B, MNS1, and SFRP4 were significantly correlated with ROS generation. *p < 0.05, **p < 0.01, and ***P < 0.001.

The signaling pathways associated with the signature genes were analyzed using ssGSEA. ECM2, METTL7B, MNS1, and SFRP4 were significantly correlated with ROS generation (**Figure 6**). Subsequently, these four genes were intersected with the oxidative stress–related genes screened in the GeneGard database, and METTL7B was finally chosen for further analysis.

### 3.7 The potential roles of METTL7B in lung cancer

**Figure 7:**
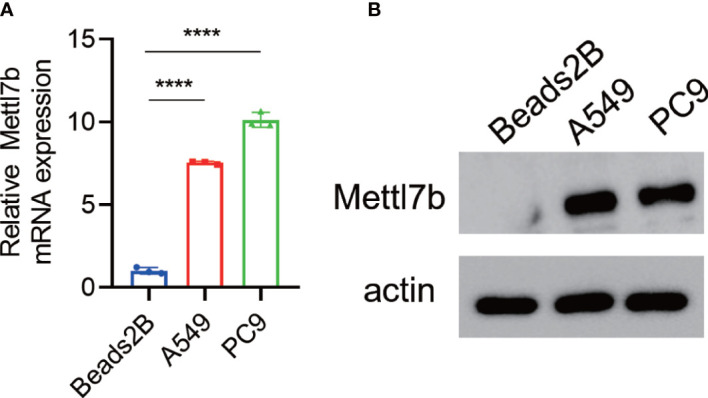
Overexpression of METTL7B in lung cancer cells. METTL7B mRNA **(A)** and protein levels **(B)** were increased in the A549 and PC9 cells. **** p<0.0001.

To investigate the roles of METTL7B in lung cancer, we first measured and compared the expression levels of METTL7B in lung cancer cell lines and normal human lung epithelial cells using qRT-PCR and Western blotting. Both the mRNA and protein expression levels of METTL7B were significantly higher in the two lung cell lines (A549 and PC9) than in the normal human lung epithelial cells (BEAS2B) (**Figures 7A, B**).

### 3.8 METTL7B is involved in ROS generation in lung cancer cells

**Figure 8:**
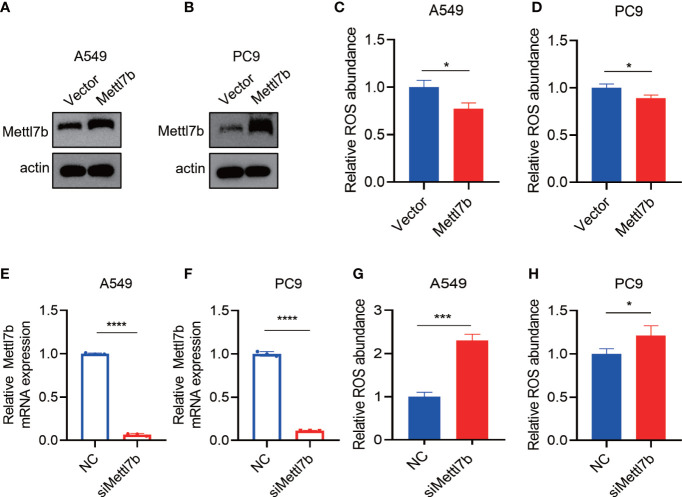
The METTL7B expression level is related to ROS generation. **(A–D)** After METTL7B was overexpressed in the A549 and PC9 cells, the relative ROS abundance was reduced. **(E–H)** mRNA was knocked down in the A549 and PC9 cells, and the relative ROS abundance was increased. * p<0.05, *** p<0.001, **** p<0.0001.

The overexpression of METTL7B protein decreased the levels of intracellular ROS (**Figures 8A–D**). By contrast, METTL7B protein knockdown increased the levels of intracellular ROS (**Figures 8E–H**).

## 4 Discussion

HF and cancer share many risk factors and epidemiological characteristics, such as high blood pressure, diabetes, obesity, smoking, unhealthy diet, and lack of physical activity. In addition, the two diseases may have some common triggering mechanisms, such as increased oxidative stress, low-level inflammatory responses, neurohormonal system activation, immune responses, and other pathological processes that can simultaneously promote the occurrence and development of HF and cancer. The relationship between the co-occurrence of HF and tumor and the mechanisms through which the two are related is not fully understood.

In this study, we tried to explore the signature genes involved in HF and investigate their potential roles in lung cancer using multiple bioinformatic techniques, including WGCNA, the LASSO algorithm, and the RF method. Four signature genes—ECM2, METTL7B, MNS1, and SFRP4—were selected and verified.

ECM2 protein is a member of the secreted protein acidic and rich in cysteine family; the proteins contained in this family are mainly related to various biological processes in the ECM (28). ECM2 gene is located in chromosome 5 (28), whereas ECM2 protein is expressed in various tissues, including the heart, brain, adrenal gland, epididymis, muscle, and lungs (29). The biological functions of ECM2 are unclear, and it is currently known to be associated with matrix assembly and cell adhesiveness (30, 31). ECM2 shares various similarities with the ECM protein. ECM2 remodeling is a key pathologic feature of HF; it is continuous and contributes to systolic and diastolic impairments (32).

*METTL7B* gene, also known as associated with lipid droplet protein 1, is located on chromosome 12q13.2. METTL7B protein belongs to the methyltransferase-like family (33) and contains a methyltransferase domain. METTL7B is involved in various diseases such as sepsis (34), lipid metabolism in non-alcoholic steatohepatitis (35, 36), and several tumors, including breast cancer (37), thyroid cancer (38), lung adenocarcinoma (39), and non–small cell lung cancer (40). Interestingly, of all these malignant diseases, the roles of METTL7B have been most widely reported in lung cancers. Liu et al. have reported that METTL7B is required for proliferation and tumorigenesis in non–small cell lung cancer (40). Li et al. have reported that METTL7B is a biomarker for prognosis and promotes the metastasis of lung adenocarcinoma cells (41). Moreover, METTL7B is mainly involved in regulating immunity and ROS generation, which are two important biological processes in both HF and lung cancer (42). In detail, METTL7B is not only a prognosis biomarker but is also involved in the tumorigenesis, proliferation (31) and metastasis of various lung cancers (32), making it a promising therapeutic target for different lung cancer subtypes (30). Therefore, we selected METTL7B for conducting an in-depth study of its potential role in the co-existence of HF and lung cancer. Our results showed that the METTL7B mRNA and protein levels were increased in two lung cancer cell lines (A549 and PC9 cells). Furthermore, after METTL7B was overexpressed in the A549 and PC9 cells, the relative ROS abundance was reduced. By contrast, METTL7B mRNA was knocked down in the A549 and PC9 cells, and the relative ROS abundance was increased. These results indicated that METTL7B may play a role in the co-occurrence of HF and lung cancer by affecting ROS-related pathways, which may act as an alternative target.

The MNS1 gene, located in chromosome 15, may play a role in the control of meiotic division and germ cell differentiation by regulating pairing and recombination during meiosis (43). MNS1 mutations are associated with the occurrence of situs inversus and male infertility (43, 44). Interestingly, recent research has shown that MNS1 may be used as a diagnostic variable when studying HF (26, 45). In 2022, Jiang et al. identified that the high expression of the MNS1 gene, together with fras1-related extracellular matrix 1 (FREM1), may affect the progression of HF by regulating bile acid, fatty acid, and heme metabolism (26). Later in the same year, by integrating three machine learning algorithms, Jiang et al. reported that FREM1 and MNS1 are diagnostic gene signatures for HF (45).

SFRP4 belongs to the SFRP family (46), which functions as soluble modulators of Wnt signaling (47). In detail, SFRP4 harbors a cysteine-rich domain homologous to the putative Wnt-binding site. The expression of SFRP4 in the ventricular myocardium is correlated with the expression of apoptosis-related genes. sFRP1-4 is expressed in cardiomyocytes, and the levels of sFRP3 and sFRP4 are elevated during HF (48).

Myocardial cells are supported by a matrix composed of vascular smooth muscle cells, endothelial cells, fibroblasts, immune cells, and their secreted cytokines and proteins. Changes in the microenvironment not only cause pathological changes such as myocardial cell hypertrophy and abnormal energy metabolism but also indirectly stimulate other organs such as tumor tissues through the action of paracrine or endocrine growth factors, cytokines, and chemokines through blood circulation (10, 49). ssGSEA analysis was conducted to analyze the immune cell infiltration between the HF and healthy groups and the correlation with the signature genes and related signaling pathways. Immune cells are involved in necrotic tissue clearance and infarct repair after myocardial infarction; they are also involved in the development of HF after myocardial infarction. In our study, the patients with HF were significantly prone to cytolytic activity, human leukocyte antigen activation, inflammation promotion, T-cell co-stimulation, and type I interferon response. Moreover, the patients with HF displayed low infiltration of B cells, macrophages, plasmacytoid dendritic cells, and Tfh and Treg cells. Treg cells are beneficial to the heart; they inhibit excessive inflammatory responses and promote stable scarring in the early stages of heart injury. However, the phenotype and function of Treg cells are altered in chronic HF (50). Moreover, different macrophage phenotypes have disparate roles in cardiovascular disease. Theoretically, manipulating macrophage phenotypes may be a means of regulating inflammation in the progression of HF (51).

Similarly, the tumor stroma is also composed of a large number of fibroblasts, lymphocytes, and macrophages and forms a complex molecular environment through the vigorous synthesis and secretion of a large number of protein components, microvesicles, exosomes, and miRNAs transported by them (52). Changes in the tumor microenvironment matrix composition provide the “soil” for the malignant growth of tumor cells; moreover, the rigid ECM (fibrosis) formed around and throughout the tumor creates a physical barrier that limits the spread of drugs to cancer cells and participates in the development of drug resistance (53). Bioinformatics and proteomics approaches have great potential for use in studying the composition of and dynamic changes in the microenvironment in HF and tumor tissues. The prevention and improvement of ECM remodeling and active tumor matrix formation are also key to the successful treatment of HF and tumors.

Our study has several limitations. First, small samples were obtained from public databases, which may lead to selection bias. Therefore, an external validation database should be used to improve the reliability of the results. Clinical trials with a larger sample size are necessary for further validation. Second, the molecular mechanisms underlying the roles of METTL7B in lung cancer and HF by regulating ROS and immune responses should be explored through more biological experiments.

## 5 Conclusions and limitations

Taken together, we screened four signature genes–ECM2, METTL7B, MNS1, and SFRP4–that may assist in the early diagnosis of HF. Furthermore, immune cell infiltration in patients with HF and their association with the gene signatures were also investigated to find clues about immunity in patients with HF. Because METTL7B gene affects the development and progression of lung cancer, its role in lung cancer cell lines has been verified. Our study has great clinical significance. First, because of the stable incidence of cancer and the improved survival rate of patients with HF, cardiologists will more often encounter patients with HF with cancer symptoms and diagnosed cancers. Second, our study provides some clues about selecting patients, particularly elderly patients with HF, with a high risk of developing cancer.

Our study has several limitations. First, whether METTL7B is uniquely and aberrantly expressed in lung cancer or is universally abnormal in pan-cancers has not yet been explored. Thus, the expression pattern of METTL7B in cancer datasets should be further validated. Second, the score of each signaling pathway in the ssGSEA of lung cancers should be further investigated. Finally, the relationship between the expression levels of METTL7B and oxidative stress should be investigated in myocardial cells.

## Data availability statement

The datasets presented in this study can be found in online repositories. The names of the repository/repositories and accession number(s) can be found within the article/**Supplementary Materials**.

## Author contributions

RD, KY, and JR designed and analyzed the research study; RD, YL, YS, and JZ wrote and revised the manuscript; YS and JZ collected and analyzed the data. All authors contributed to the article and approved the submitted version.

## Supplementary material

Supplementary Figure 1 The research flow chart.
