# Supplementary material for: Heart failure–related genes associated with oxidative stress and the immune landscape in lung cancer
Source: Front Immunol. 2023 May 18;14:1167446. doi: 10.3389/fimmu.2023.1167446 (PMC10232804; doi:10.3389/fimmu.2023.1167446)
Supplement: Supplementary file 1 [file Table_1.doc]

**Table S1 Primers used in this study.**

| GAPDH forward | 5'- ACAACTTTGGTATCGTGGAAGG-3' |
| --- | --- |
| GAPDH reverse | 5'- GCCATCACGCCACAGTTTC-3' |
| METTL7B forward | 5'-GCAACCGCAAGATGGAGAG-3' |
| METTL7B reverse | 5'-GATTTGGGTCTAGGCAGGTGA-3' |
