# Supplementary figures and images for: Heart failure–related genes associated with oxidative stress and the immune landscape in lung cancer
Source: Front Immunol. 2023 May 18;14:1167446. doi: 10.3389/fimmu.2023.1167446 (PMC10232804; doi:10.3389/fimmu.2023.1167446)

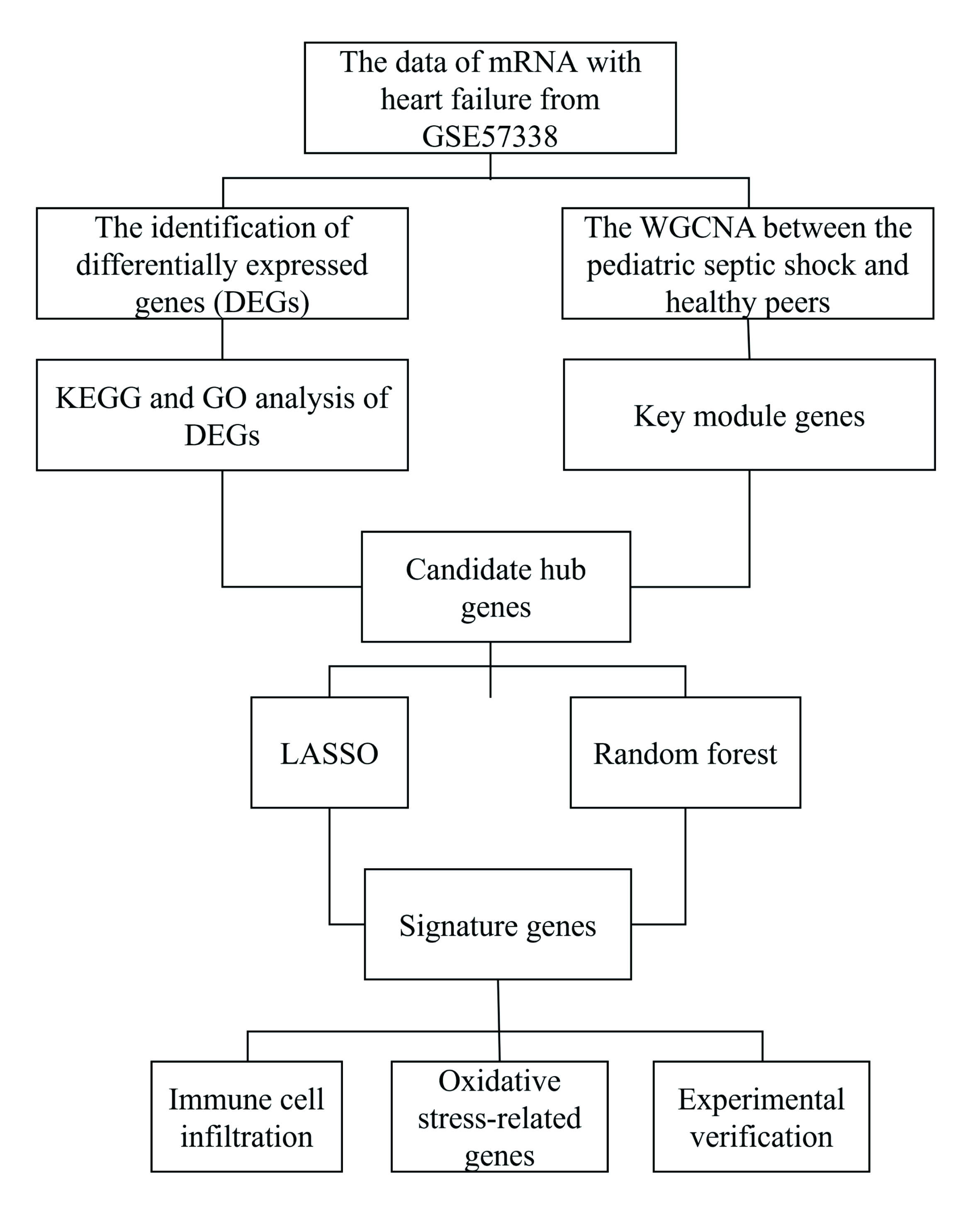

Supplement: Supplementary Figure 1 — The research flow chart. [file Image_1.tif]
